# Corrections

**Published:** 2004-12

In the letter by Storm and Mazor [Environ Health Perspect 112:A862–A864 (2004)], the title of Table 1 was incorrect; also, P1 and P2 were not exposed but were unexposed children tested in another study. The corrected table is presented below.

At the time the October 2004 Forum article “Farm Chore Checkup” [Environ Health Perspect 112:A804 (2004)] went to press, Anne Gadomski’s assessment of the North American Guidelines for Children’s Agricultural Tasks was scheduled for publication in the October 2004 issue of the *American Journal of Public Health* (*AJPH*). However, publication of the assessment in *AJPH* was delayed; a new publication date has not been set. *EHP* regrets the error.

Wasserman et al. detected errors in their article “Water Arsenic Exposure and Children’s Intellectual Function in Araihazar, Bangladesh” [Environ Health Perspect 112:1329–1333 (2004)]. In the first paragraph of “Results” (p. 1331), the values should be reversed to read “On average, mothers and fathers reported 2.9 and 3.7 years of education, respectively.” In the second paragraph of “Results” (“Exposure characteristics”), the mean water As concentration should be 117.8 μg/L, not 117.8 μg/dL.

Also, on page 1332 in “As metabolism,” the authors would like to clarify that Chowdury et al. (2003) reported that only the first reaction of the arsenic metabolic pathway—the formation of MMA—is less active in children than in adults.

## Figures and Tables

**Table 1:** Child participants in VCS studies.

Exposed			Unexposed		
ID	Age	DD or ADD	ID	Age	DD or ADD
E9*^a^*	8	–	C9*^a^*	9	–
E10*^a^*,*^b^*	6	X	C10*^a^*	8	–
E14*^a^*,*^b^*	12	X	C14*^a^*	12	–
E17*^a^*	6	–	C17*^a^*	5,7	–
			P1*^b^*	8	X
			P2*^b^*	10	X

a Children shown in Figure 1A (NYSDOH, unpublished data; Schreiber et al. 2002).

b Children shown in Figure 1B: E10 and E14 were from Schreiber et al. (2002); P1 and P2 were unexposed children examined in an NYSDOH study (NYSDOH 2004).

